# The role of IL-37 and IL-38 in rheumatoid arthritis, the potential clinical applications in precision medicine

**DOI:** 10.3389/fimmu.2025.1629759

**Published:** 2025-07-24

**Authors:** Haifeng Zhang, Yuna Ye, Xingyan Ling, Brett D. Hambly, Shisan Bao

**Affiliations:** ^1^ The Cardiovascular Centre, The First People’s Hospital of Baiyin, Baiyin, Gansu, China; ^2^ Department of Stomatology, The First People’s Hospital of Baiyin, Baiyin, Gansu, China; ^3^ Scientific Research Division, The First People’s Hospital of Baiyin, Baiyin, Gansu, China

**Keywords:** IL-37, IL-38, rheumatoid arthritis, precision medicine, pathogenesis

## Abstract

Rheumatoid arthritis (RA) is a chronic, autoimmune inflammatory disorder that primarily affects the joints, and in severe cases, can damage other major organs, particularly in susceptible individuals. Management of RA primarily relies on disease-modifying anti-rheumatic drugs (DMARDs) often used in conjunction with low-dose steroids; however, outcomes are frequently suboptimal, resulting in significant physical and psychological impact. Biological agents have shown promise for non-responsive RA patients. Nevertheless, the precise underlying mechanism of RA remains unclear. Systemic and local levels of IL-37 and IL-38, anti-inflammatory cytokines, are elevated in RA patients. Intriguingly, these levels decrease in individuals experiencing remission, correlating with the Disease Activity Score (DAS28) and histopathological findings. In animal models, exogenous IL-37 and IL-38 demonstrate protective effects against RA development, while depletion of either cytokine exacerbates the disease *in vivo*. These findings suggest that the elevated IL-37 and IL-38 represent a compensatory response to the substantial inflammation in affected joints, attempting to mitigate dysregulated host immunity, albeit unsuccessfully. These data offer potential insights for developing novel, more effective RA therapies through precision medicine approaches.

## Rheumatoid arthritis

Rheumatoid arthritis (RA) is a chronic inflammatory disorder caused by autoimmune dysfunction and primarily affects the joints (1). Although the exact aetiology remains unclear, RA is believed to result from immune dysregulation triggered by environmental factors in genetically predisposed individuals. Certain alleles—such as *HLA-DRB1*, *DRB1*, *DRB1*, and *DRB1*—have been associated with increased susceptibility to RA (2). While RA predominantly targets joint cartilage (3), it can also involve extra-articular organs, including the skin, eyes, lungs, heart, and blood vessels (4). Early diagnosis is crucial for halting or delaying disease progression through timely intervention aimed at achieving remission (5).

Treatment strategies for RA aim to induce remission or reduce disease activity. Disease-modifying anti-rheumatic drugs (DMARDs), such as methotrexate, are commonly prescribed in combination with short-term, low-dose glucocorticoids to broadly suppress immune responses mediated by T cells, B cells, and monocytes/macrophages (1, 6). While many patients respond well to initial treatment, a subset fails to achieve remission and requires second-line therapies, such as biological agents targeting TNF, IL-6 receptors, and CD80 (7). However, long-term use of monoclonal antibodies is associated with undesirable adverse effects, including allergic reactions and increased malignancy risk (8). In this context, Traditional Chinese Medicine (TCM), particularly in China, has shown potential in reducing side effects and enhancing therapeutic outcomes (9). Despite advances in treatment, severe RA can still lead to physical disability and remains a significant clinical challenge (6).

Clinically, RA often presents with tender, swollen joints—particularly in the fingers—as well as fatigue, fever, and loss of appetite (10) likely reflecting systemic immune dysregulation (11). Disease activity is commonly assessed using the Disease Activity Score 28 (DAS28), which evaluates 28 joints based on swelling, tenderness, patient global assessment, and erythrocyte sedimentation rate (ESR) (12).

The pathogenesis of RA involves synovial fibroblast hyperplasia and infiltration of the synovial membrane by T and B lymphocytes and macrophages, leading to cartilage destruction and bone erosion (6, 13). These immune cells secrete pro-inflammatory cytokines—such as IL-6, IL-1β, and TNF—that sustain a chronic inflammatory environment (11).

**Figure 1:**
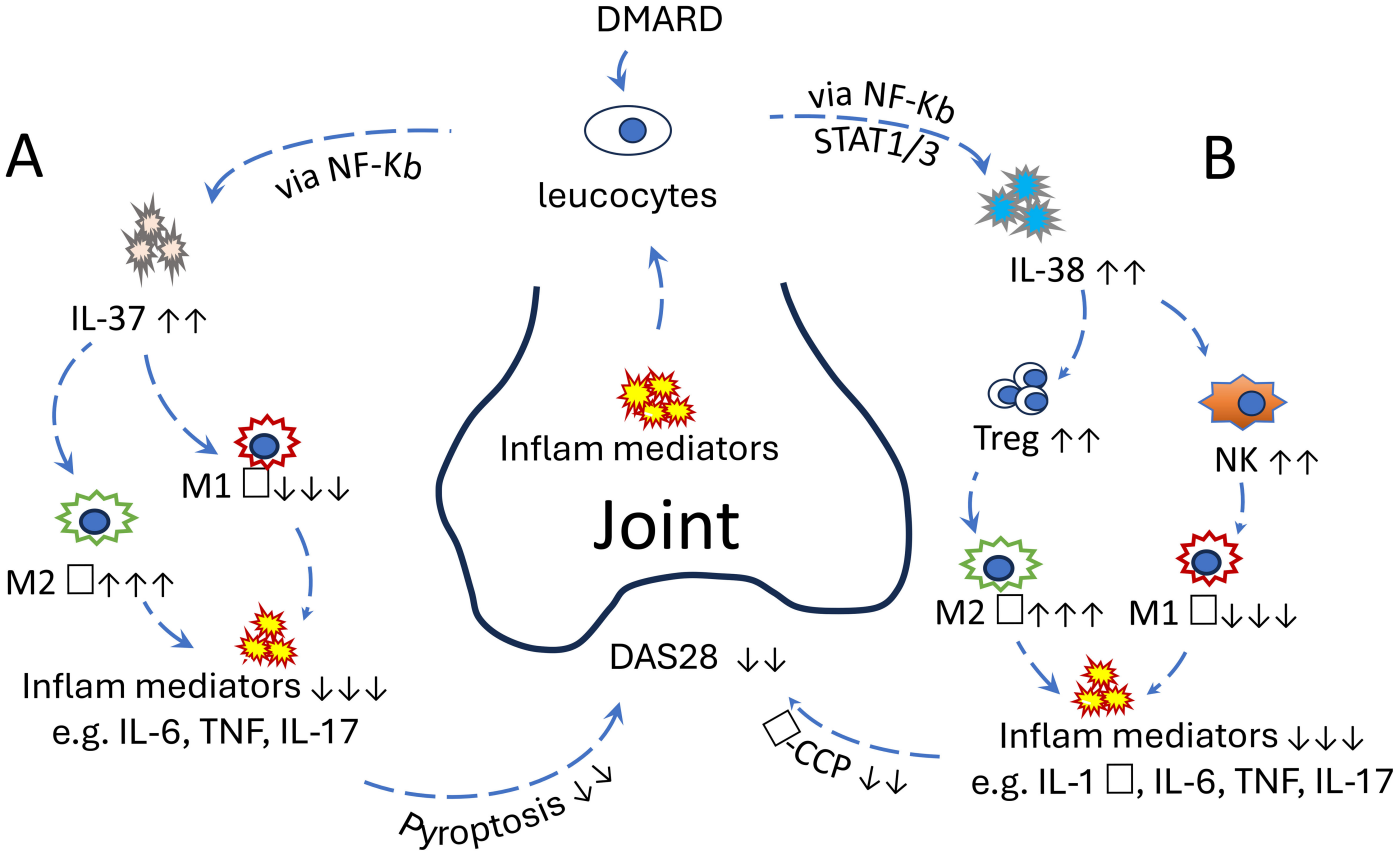
**(A)** The protective role of IL-37 in RA: Increased proinflammatory mediators in the joints of RA patients stimulate IL-37 secretion from leucocytes *via* the NF-κB pathway. IL-37 subsequently promotes the polarisation of M2 macrophages while inhibiting M1 macrophages. This anti-inflammatory response reduces proinflammatory cytokine levels and pyroptosis in joint cells, ultimately leading to a decreased DAS28 score. This effect can be further enhanced by the addition of disease-modifying antirheumatic drugs. **(B)** The protective role of IL-38 in RA. Increased proinflammatory mediators in the joints of RA patients stimulate IL-38 secretion from leucocytes *via* STAT1, STAT3, p38 MAPK, ERK1/2, and NF-κB. IL-38 subsequently promotes the proliferation of Treg and NK cells, followed by the polarisation of M2 macrophages and inhibition of M1 macrophages. Consequently, anti-inflammatory response reduces proinflammatory cytokine levels in joint tissues, ultimately leading to a decreased DAS28 score. The effect can be further enhanced by the addition of disease-modifying antirheumatic drugs. These clinical manifestations could also be improved with exogenous IL-37 or IL-38 *in vivo*, suggesting their potential as promising therapeutic targets.

Understanding the immunological mechanisms underlying RA is crucial for developing more effective therapies. This mini-review focuses on the roles of IL-37 and IL-38 in RA pathogenesis, particularly in relation to macrophage polarisation, and explores their potential as therapeutic targets in precision medicine. While IL-36, IL-37, and IL-38 have been broadly investigated in both osteoarthritis (OA) and RA (14), this review examines IL-37 and IL-38 in RA more specifically, highlighting their emerging roles and therapeutic promise (**Figure 1**).

## IL-37 and IL-38

IL-37 and IL-38, members of the IL-1 superfamily (15), are key regulators of immune homeostasis. Although structurally related to pro-inflammatory IL-1 family cytokines, both IL-37 and IL-38 exhibit predominantly anti-inflammatory properties and are expressed across a broad range of organs and tissues. This widespread expression underscores their potential for systemic influence on immune regulation.

Notably, IL-37 and IL-38 are expressed in immune cells such as natural killer (NK) cells, B lymphocytes, and monocytes, suggesting direct roles in modulating immune cell function (16). Their presence in barrier tissues—including keratinocytes and epithelial cells—also indicates a role in maintaining local immune balance at sites of pathogen entry or environmental exposure (17). In addition, these cytokines are found in parenchymal organs such as the heart, lungs, intestines, urogenital system, and skin (18), as well as in secondary lymphoid tissues like the spleen and tonsils (19), highlighting their broad involvement in tissue homeostasis and inflammatory regulation across diverse physiological settings.

IL-37 attenuates inflammation by suppressing both innate and adaptive immune responses (20), including antigen-specific responses of the adaptive immune system (21). This dual suppression leads to an overall dampening of host immune reactivity (20), which can be protective against excessive inflammation (22) but must be tightly regulated to avoid compromising pathogen defence. The immunoregulatory effects of IL-37 have been implicated in a range of chronic inflammatory and immune-mediated diseases. In cancer, IL-37 may influence the tumour microenvironment and has been reported to suppress tumour growth under certain conditions (23). In inflammatory bowel diseases, such as Crohn’s disease, IL-37 helps modulate excessive immune responses contributing to intestinal inflammation (23). Pre-clinical studies in IL-37 transgenic animals and human data further support its athero-protective roles, including reduced development of atherosclerotic lesions and decreased atheroma formation (24), often accompanied by increased production of other anti-inflammatory mediators that facilitate vascular inflammation resolution.

IL-38 also plays a critical role in immune regulation by helping to balance pro- and anti-inflammatory responses (25). A unique aspect of IL-38 biology is its release from apoptotic cells, suggesting a role in dampening inflammation during tissue remodelling or cellular turnover (26). Once released, IL-38 acts directly on inflammatory macrophages—central players in innate immune activation—by suppressing their pro-inflammatory functions (26). Loss or dysfunction of IL-38 can disrupt this regulatory balance, contributing to a pro-inflammatory microenvironment and promoting disease development and progression (25), e.g. in viral infection (27) or psoriasis (28).

Mechanistically, IL-38 downregulates the production of key pro-inflammatory cytokines, including IL-6, TNF, CCL5, and CXCL10 (29). These effects are mediated through modulation of intracellular signalling pathways such as STAT1, STAT3, p38 MAPK, ERK1/2, and NF-κB (29), offering molecular insight into its anti-inflammatory action. Such regulatory effects have been demonstrated *in vivo*, including in the NOD/SCID murine model of allergic asthma (29).

### IL-37 in RA

Circulating IL-37 is significantly upregulated in patients with RA compared to healthy controls (30, 31), with levels correlating positively with disease severity and decreasing in patients in remission (30, 31). With an area under the curve (AUC) of 0.7789, IL-37 also shows promise as a diagnostic biomarker for RA, further supporting its involvement in disease development (30, 31). These findings implicate IL-37 in RA pathogenesis, although the precise mechanisms driving its upregulation remain unclear. As an anti-inflammatory cytokine, elevated IL-37 levels may reflect a compensatory response to heightened systemic inflammation—mirrored by increased CCP, ESR, and IL-17 levels (32). However, in genetically or environmentally predisposed individuals, this response may be inadequate (33), particularly when persistent inflammatory triggers such as tobacco use sustain synovial inflammation and joint damage (33).

To further validate IL-37 expression, mRNA levels have been measured in synovial cells and peripheral blood mononuclear cells (PBMCs) from RA patients. Synovial cell mRNA is considered a more accurate marker of local immune activity than circulating cytokine levels. IL-37 mRNA is significantly upregulated in both synovial cells and PBMCs and correlates with high DAS28 scores (5–9) (30, 31). Immunohistochemical analysis further confirms increased IL-37 expression in RA synovial tissue relative to healthy controls (34). These findings are supported by other studies reporting elevated IL-37 mRNA and protein levels in PBMCs of patients with active RA, while no significant differences are observed in those in remission (35). This likely reflects successful inflammatory control. Notably, circulating IL-37 decreases following glucocorticoid treatment and correlates with reductions in CRP, ESR, and disease activity (35).

Given the central role of Th17 cells and IL-17 in RA pathogenesis (36), the relationship between IL-37 and IL-17 has also been examined. IL-37 inhibits IL-17 expression in CD4^+^ T cells from RA patients and reduces Th17 polarisation *in vitro* following LPS and PMA stimulation, suggesting that IL-37 may regulate aberrant immunity *via* Th17 cell-driven IL-17 production (35). Additionally, plasma IL-37 levels positively correlate with IL-17A, TNF, and DAS28 scores, and are modulated in response to DMARD therapy (37). These associations merit further validation *in vivo* and in human samples, particularly through joint biopsies and PBMC analysis in patients receiving DMARDs and/or biological therapies (1).

Despite its anti-inflammatory properties, the elevation of IL-37 in RA presents a paradox. IL-37 is produced mainly by CD3^+^ and CD4^+^ T cells and functions both intra- and extra-cellularly (38). One proposed mechanism involves inhibition of the TNF-mediated NF-κB/Gasdermin D (GSDMD) signalling pathway, thereby reducing pyroptosis in fibroblast-like synoviocytes (39), although the requirement for NF-κB activation in GSDMD-mediated pyroptosis remains uncertain.

Supporting its anti-inflammatory function, recombinant human IL-37 (rhIL-37) suppresses IL-17, IL-1β, and IL-6 production in PBMCs from healthy individuals following inflammatory stimulation (35). *In vivo*, intra-articular administration of rhIL-37 in collagen-induced arthritis (CIA) mice reduces disease severity and local cytokine expression. Similarly, rhIL-37 downregulates pro-inflammatory cytokines in murine macrophage cell lines, and IL-37 transgenic mice show reduced LPS-induced inflammation *via* SMAD signalling (34). Altogether, while IL-37 elevation in RA may represent an endogenous attempt to counteract inflammation, this response may be insufficient in susceptible individuals. Nonetheless, its downregulation following effective therapy supports a role in disease modulation and progression, highlighting IL-37 as a potential therapeutic target in RA.

Taken together, current evidence suggests that IL-37 expression in both the systemic circulation and joint tissue reflects a host attempt to suppress chronic inflammation. In individuals with high inflammatory burden or reduced regulatory capacity, this response may be overwhelmed. Nevertheless, the continued expression of IL-37—and of IL-38, discussed below—suggests an ongoing immunological effort to contain disease activity.

From an immunological perspective, IL-37 may also influence RA pathogenesis by modulating macrophage polarisation, specifically the balance between pro-inflammatory (M1) and anti-inflammatory (M2) subsets (22, 40). Although direct evidence in RA is limited, recombinant IL-37 reduces pro-inflammatory cytokine production in macrophages (41). In calcified heart valves, IL-37 expression correlates positively with the presence of M2 and negatively with M1 macrophages, suggesting it may suppress M1 polarisation *via* the Notch1 pathway (42). Further studies are needed to clarify IL-37’s role in macrophage regulation during RA, which are discussed in the final section.

### IL-38 in RA

Studies have shown significantly elevated circulating IL-38 levels in patients with RA compared to healthy controls, with higher levels correlating with increased disease activity, as measured by DAS28 scores and objectively, using ultrasonography, further supporting these clinical associations (43). This clinical presentation is accompanied by a marked upregulation of circulating IL-38, along with elevated levels of pro-inflammatory cytokines such as IL-1β, IL-17, IL-6, and TNF. Moreover, analyses of plasma IL-38 protein and PBMC mRNA levels have revealed a significant correlation between IL-38 mRNA and protein expression, confirming the consistency of its upregulation in active RA (43, 44). This pattern likely reflects the host’s attempt to counteract persistent local and systemic inflammation in patients with active disease. Notably, elevated IL-38 levels, together with those of pro-inflammatory mediators, show strong correlations with DAS28 scores, swollen joint count, and tender joint count (43, 44). These observations suggest that increased IL-38 expression may represent a compensatory—though ultimately insufficient—response to ongoing inflammation, particularly in genetically or environmentally predisposed individuals.

Beyond these clinical associations, IL-38 also shows promise as a diagnostic marker. An AUC value of 0.84 supports its potential utility not only in reflecting disease activity and therapeutic response but also as a possible therapeutic target (43, 44). The elevated IL-38 levels observed in RA patients—paralleling the compensatory IL-37 response discussed earlier—may indicate an endogenous anti-inflammatory mechanism that becomes inadequate in severe or treatment-resistant cases.

Supporting this hypothesis, IL-38 levels are significantly higher in RA patients who test positive for anti-cyclic citrullinated peptide (anti-CCP) antibodies compared to seronegative individuals, aligning with the established diagnostic and prognostic value of anti-CCP (43, 44). Moreover, IL-38 levels decline following six months of treatment with DMARDs, reinforcing its association with disease activity and treatment response (43, 44). Its differential expression between RA and OA further underscores its specific involvement in immune-mediated joint pathology (14).

While these findings are based on systemic measurements—likely reflecting IL-38 production by M2 macrophages (22) - direct evidence from the site of inflammation remains scarce. Synovial biopsies from RA patients would provide more specific insight, particularly if collected longitudinally before and after treatment. However, such sampling is often limited by ethical and practical constraints. An alternative strategy involves analysing joint tissues from RA cohorts using post-mortem human samples obtained from morgue collections, as previously described in studies of heart tissue (45, 46). This approach may provide more objective and temporally informative data, particularly in relation to therapeutic interventions and their correlation with disease history. This approach could be enhanced by advanced histopathological and multiplex immunohistochemical techniques (47), providing valuable mechanistic insight into the pathogenesis of RA.

Together, these data support the concept that RA progression involves a complex interplay between pro- and anti-inflammatory responses. IL-38 upregulation likely reflects an intrinsic effort to counterbalance inflammation. However, in individuals with persistent or treatment-refractory disease, this compensatory mechanism may fail to control disease progression. It remains unclear whether IL-38 exerts its effects in an autocrine or paracrine manner within inflamed joint tissues (48). Addressing this question will require spatial immunophenotyping, such as multiplex immunostaining (47), to localise M1 and M2 macrophages and other immune cell populations in the synovium under various disease states to further investigate the underlying immunological mechanism.

Further supporting IL-38’s local role, studies have shown that IL-38 is significantly upregulated in the synovial tissue of RA patients, as demonstrated by immunohistochemistry and ELISA (49). While Liang et al. (43), reported elevated circulating IL-38 levels in RA, Takenaka et al. found no significant differences among RA, OA, and healthy controls in the circulation. These discrepancies may be due to differences in methodology, sample size, or patient demographics. Nonetheless, synovial IL-38 appears to better reflect local disease activity, especially during active flares, and its expression diminishes during remission (43), reinforcing its putative anti-inflammatory role.

The protective function of IL-38 has also been demonstrated in animal models. In experimental arthritis, IL-38 expression increases in the arthritic joints of wild-type (WT) mice, consistent with findings in human RA (43, 44, 49). In contrast, IL-38 knockout (KO) mice exhibit more severe disease, with higher clinical scores, greater histopathological damage, and increased levels of IL-6 and IL-1β in joint tissues (49). These findings confirm a protective role for IL-38, particularly in the early stages of arthritis in the animal model. Furthermore, administration of rIL-38 in these models alleviates disease severity (48), characterised by reduced M1 macrophage infiltration and decreased production of Th17-associated cytokines—highlighting its regulatory effect through differential macrophage polarisation and subsequent cytokine production. Although the precise role of IL-38 in macrophage polarisation within RA remains to be fully clarified, emerging evidence suggests that IL-38 promotes the transition from M1 to M2 macrophages (22) by inhibiting NLRP3 inflammasome activation and promoting anti-inflammatory cytokine release (50).

Further investigations using both animal models and human tissues are needed to better elucidate the immunomodulatory functions of IL-38 and its potential as a therapeutic target in RA. Despite the IL-38 KO animal RA model having shown its anti-inflammatory benefits, caution is warranted when translating findings from murine models to human RA. Additionally, differences in body size, disease chronicity, and treatment protocols limit the direct applicability of animal data to the human condition. Moreover, IL-38 overexpression in mice does not significantly prevent cartilage or bone destruction, likely due to the shorter disease duration in animal models compared to the chronic progression of human RA (43, 44). Nevertheless, *in vitro* studies provide additional mechanistic insights: IL-38 overexpression reduces IL-6, TNF, and IL-23 production in THP-1 monocytic cells. Similar anti-inflammatory effects have been observed in primary macrophages and synovial fibroblasts derived from RA patients (43, 44). These findings are consistent with earlier reports that IL-38 suppresses *Candida albicans*-induced IL-17 production in PBMCs (51).

At the signalling level, IL-38 exerts its effects *via* the IL-36 receptor (IL-36R), where it inhibits recruitment of the IL-1 receptor accessory protein (IL-1RAcP), potentially favouring the binding of inhibitory receptor complexes instead (43, 44), This disruption blocks MyD88 activation, thereby attenuating downstream NF-κB and MAPK signalling pathways and suppressing inflammatory cytokine production (52).

## Future developmental strategies in IL-37 and/or IL-38 application in RA

To investigate the therapeutic potential, safety, and translational feasibility of recombinant human IL-37 and IL-38 (rhIL-37/38) in RA, we propose a comprehensive two-stage experimental framework integrating *in vitro*, *in vivo*, and *ex vivo* approaches.

### Stage one: *in vitro* functional characterisation

The efficacy and cytotoxicity of rhIL-37 and/or rhIL-38 *in vitro* will be evaluated using human primary monocytes isolated from peripheral blood mononuclear cells (PBMCs) or fibroblasts, with source protocols based on established methods (53). A key objective is to assess the capacity of these cytokines to modulate macrophage polarisation—promoting anti-inflammatory M2 differentiation while inhibiting pro-inflammatory M1 polarisation—under inflammatory conditions such as lipopolysaccharide stimulation.

Functional readouts will include proliferation (Ki67 expression), apoptosis (*via* flow cytometry using markers such as Annexin V or PI), and cytokine profiling of the supernatants using ELISA to quantify both pro- and anti-inflammatory mediators. Cytotoxicity will be systematically assessed through titration-based assays including Hoechst 33342 and propidium iodide staining (54). These studies will be conducted in parallel on PBMCs from both RA patients and healthy controls to identify optimal inhibitory concentrations with minimal off-target effects.

### Stage two: *in vivo* and ex vivo validation

Following optimisation of dosage and exposure time *in vitro*, *in vivo studies* will assess the pharmacokinetics, therapeutic efficacy, and systemic safety of exogenously administered IL-37 and IL-38 in RA animal models (43, 44). Methodology will involve the use of multiplex immunohistochemistry (47) to quantify and localise M1 and M2 macrophages in RA tissue (22), assess their interaction with other immune subsets, and evaluate changes under various conditions. Such experiments should be extended to human RA samples for translational relevance. This may yield valuable insights into the development of safer and more effective biologics for RA treatment. These experiments will aim to define the minimal effective dose and treatment window for maximal suppression of disease progression. Safety profiling will involve histopathological examination of major organs and imaging-based evaluation of therapeutic outcomes (55).

*Ex vivo validation* will subsequently be performed using human biopsy or resected joint tissue samples to confirm cytokine bioactivity in a more clinically relevant environment (56). All studies involving human-derived samples will be conducted under approved ethical guidelines.

### Translational considerations: delivery and clinical applicability

Although IL-37 has shown protective roles in RA, its pharmacokinetics *in vivo* remain poorly characterised, and it is unclear whether endogenous IL-37 or IL-38 can be sufficiently upregulated in response to immunogenic triggers. Therefore, effective delivery strategies are critical. These may include gene gun-mediated expression (57), recombinant protein administration (58), or viral vector-based systems (59). Each method will be comparatively assessed for safety, bioactivity, and tissue penetration, beginning with *in vitro* testing, followed by *in vivo* validation in animal models (60), and *ex vivo* studies in human tissues (61). Emerging localised delivery platforms such as hydrogels also show promise for enhancing IL-38 bioavailability in arthritic joints and warrant further exploration (62). These investigations will inform the selection of the most appropriate delivery platform for therapeutic application.

### Biomarker potential and clinical translation

Initial evidence suggests that IL-37 and IL-38 levels in PBMC —both in serum and circulating leukocytes—correlate with disease severity in RA (30, 31, 43, 44). Notably, IL-38 expression in synovial tissue exhibits even stronger associations with disease activity (43, 44). These findings provide a foundation for the development of multiplexed biomarker platforms to monitor disease progression and treatment response. Such biomarkers could greatly enhance our mechanistic understanding of RA and guide personalised therapeutic strategies.

Together, this multi-tiered approach will not only establish the therapeutic viability and safety of rhIL-37/38 but also clarify optimal delivery methods, identify predictive biomarkers, and support their translational potential in the management of RA. Pre-clinical validation of these cytokines is a critical step towards future clinical trials and eventual clinical application in refractory RA.

## Conclusion

IL-37 and IL-38 contribute to immune regulation in RA, primarily by suppressing proinflammatory pathways and modulating macrophage polarisation. IL-37 acts early by inhibiting NF-κB and MAPK signalling, while IL-38 limits Th17 responses and promotes regulatory T-cell function. However, their protective effects may be insufficient in chronic or severe RA. Both cytokines represent promising therapeutic targets due to their roles in shifting macrophages from a pro- to anti-inflammatory state. Future strategies could include cytokine-based therapies, local delivery systems, or bioactive compounds from traditional Chinese medicine. To facilitate readers’ understanding, a schematic figure (**Figure 1**) has been added to illustrate the interaction of IL-37 and IL-38 with host immunity in RA patients.
